# Early and Mid-Term Outcomes of Transcaval Embolization for Type 2 Endoleak after Endovascular Aortic Repair

**DOI:** 10.3390/jcm13123578

**Published:** 2024-06-18

**Authors:** Petroula Nana, Giuseppe Panuccio, Fiona Rohlffs, José I. Torrealba, Konstantinos Spanos, Tilo Kölbel

**Affiliations:** German Aortic Center, Department of Vascular Medicine, University Heart and Vascular Center UKE Hamburg, 20246 Hamburg, Germany; g.panuccio@uke.de (G.P.); fionarohlffs@web.de (F.R.); jitorrealba@gmail.com (J.I.T.); spanos.kon@gmail.com (K.S.); tilokoelbel@googlemail.com (T.K.)

**Keywords:** transcaval embolization, endoleak, endovascular aortic repair

## Abstract

**Background**: Among the endovascular approaches for the management of endoleak type 2 (EL 2), transcaval embolization (TCE) has shown encouraging outcomes. However, the literature is still limited. This study aimed to present the early and mid-term outcomes of TCE for EL 2 after endovascular aortic repair. **Methods**: A retrospective, single-center analysis of consecutive patients managed with TCE for EL 2 after standard or complex endovascular aortic repair, from August 2015 to March 2024, was conducted. The indication for TCE was the presence of an EL 2 related to ≥5 mm sac increase, compared to the first imaging after aneurysm exclusion or the smallest diameter during follow-up. Patients managed with TCE for other types of endoleaks were excluded. The primary outcomes were technical and clinical successes during follow-up. **Results:** Forty-three patients were included (mean age: 75.1 ± 6.0 years, 90.7% males). Technical success was 97.7%. Selective embolization was performed in 48.8% and non-selective in 51.2%. No death was recorded at 30 days. The estimated clinical success was 90.0% (standard error; SE: 6.7%) and the freedom from EL 2 was 89.0% (SE 6.4%) at 36 months. Cox regression analysis showed that the type of embolization (selective vs. non-selective), type of previous repair (f/bEVAR vs. EVAR), and use of anticoagulants did not affect follow-up outcomes. Reinterventions related to EL 2 were performed in 12.5%; three underwent an open conversion. **Conclusions**: TCE was related to high technical success and limited peri-operative morbidity, regardless of the type of initial endovascular aortic repair. Clinical success was encouraging with reinterventions for EL 2 affecting 12.5% of patients.

## 1. Introduction

Endoleak type 2 (EL 2) after endovascular aortic aneurysm repair (EVAR) is considered a benign condition with self-limiting behavior in most cases [1,2]. Similarly, in patients managed with complex endovascular procedures, such as fenestrated and branched endovascular aortic aneurysm repair (f/bEVAR), EL 2 may be detected in almost 25% of patients during follow-up [3]. Specific endoleak flow characteristics and patterns, such as high and to-and-fro flow, may be able to predict persistent EL 2 and any associated adverse events, including sac expansion and aneurysm rupture [1,4]. According to the current guidelines, EL 2 should be managed with a “wait and watch” approach using adequate imaging, while intervention is indicated only when the endoleak is related to sac expansion ≥ 5 mm [5]. 

Different endovascular approaches have been used for EL 2 embolization and despite their high technical success, EL 2 recurrence is not uncommon, and many patients undergo multiple interventions for complete sac exclusion [6]. Patients with persistent EL 2 are at risk for sac and endograft instability, due to sealing zone loss after EL 2-related sac expansion [6]. Transarterial and translumbar embolization, using a variety of embolic agents, such as coils, glue, and Onyx^®^, represent the main modalities applied for EL 2 management, with comparable outcomes in terms of technical and clinical success [7,8]. Selective embolization of side branches has been related to 76% sac exclusion with the first attempt and without significant morbidity [8]. Selective pre-emptive embolization has also shown encouraging outcomes [9]. However, radiation exposure during this step should be considered [10]. Other approaches, including trans-endograft and transcaval embolization (TCE) have been also successfully applied [11,12]. However, the published experience is quite limited. Previous systematic review data on 117 patients managed with TCE showed high technical success and low persistent EL 2 rates during the 12-month follow-up period [12]. 

This study aims to present a single-center analysis on the early and mid-term outcomes of patients managed with TCE for EL 2 after endovascular aortic repair, including standard EVAR and f/bEVAR.

## 2. Materials and Methods

### 2.1. Study Design

A retrospective analysis of consecutive patients managed with TCE for EL 2 was conducted following the STROBE guidelines [13]. All patients were managed electively from August 2015 to March 2024 in a single aortic center. The indication for repair was sac expansion ≥ 5 mm due to the presence of persistent EL 2 after previous endovascular aortic repair, EVAR or f/bEVAR. The sac expansion was evaluated using the diameter of the sac after the completion of the endovascular exclusion of the aneurysm or the smallest diameter reported during follow-up. The study complied with the Declaration of Helsinki and was considered exempt from ethical approval according to the local laws, given its retrospective design and anonymized data.

### 2.2. Patient Cohort

The extent of the underlying disease was not a criterion for exclusion; patients with infra-, juxta-, para-renal, and thoracoabdominal aneurysms (TAAAs) were included. Patients managed with TCE for other types of endoleaks (two patients with type Ia) or needing concomitant embolization for any other artery than the inferior mesenteric (IMA) or lumbar arteries were excluded. In four cases, an IMA embolization was performed intentionally through the superior mesenteric artery (SMA) before the transcaval puncture. These patients were considered eligible, as a transcaval embolization was performed subsequently for non-selective sac or selective lumbar embolization, depending on the anatomy of the patient. 

Initial endovascular aortic treatment was performed either at this or another institution (two EVAR cases). For our institution, the follow-up protocol after endovascular aortic repair consisted of computed tomography angiography (CTA) before discharge, at 12 months, and yearly thereafter. However, modifications to the protocol were made upon imaging findings or the patient’s symptoms. Patients with an EL 2 detected at the pre-discharge CTA were reevaluated at 6 months. In the case that the aneurysm sac was stable in two subsequent evaluations with a time interval of 6 months, the patient was managed according to the standard follow-up protocol (yearly). In the case that sac expansion ≥5 mm was detected and depending on the aortic, contributing vessel, and vena cava anatomy, the patient was considered as a candidate for TCE. 

Contrast-enhanced ultrasound (CEUS) was used in patients with decreased renal function (estimated glomerular function rate (eGFR) < 60 mL/min) or in the case that the performed CTA could not provide adequate information on endoleak differentiation.

### 2.3. Anatomic and Technical Details

A patient was considered a candidate for TCE, if the following anatomic criteria were present: 1. The aneurysm sac was in close proximity to the vena cava in a segment allowing access to the perfused aneurysm; 2. The endoleak nidus was in proximity and accessible through a transcaval approach; 3. If a lumbar artery was the main contributing vessel for EL 2 formation. In the case of the IMA being the main contributing vessel, a transarterial approach through the SMA was chosen for the selective embolization of the artery. 

**Figure 1:**
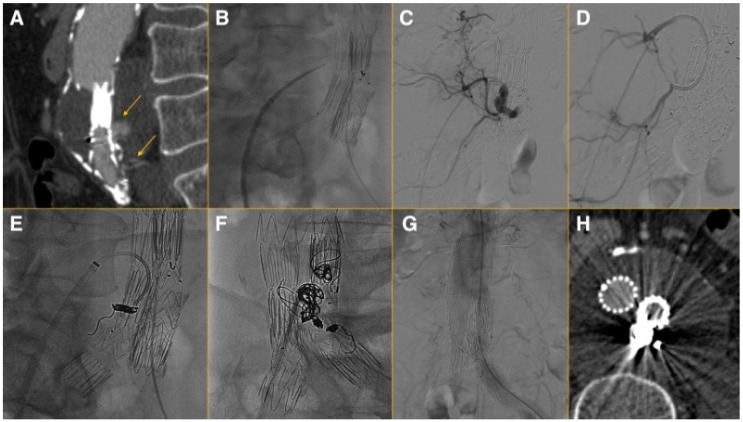
After evaluating the CTA and confirming the presence of an EL 2, especially related to the lumbar arteries (**A**) and if the patient presented with the anatomic criteria, they were considered to be a candidate for TCE under local or general anesthesia. Percutaneous arterial access from the right or left femoral artery was used for angiography to rule out other endoleaks. The right common femoral vein was used for venous access. An 8F SL0 sheath dilator system (St. Jude Medical, St. Paul, MN, USA) was positioned against the vena cava wall. A Brockenbrough needle (Medtronic, Minneapolis, MN, USA) was coaxially introduced and the aneurysm sac was then punctured (**B**). The aneurysm sac puncture site was near the endoleak nidus or contributing vessel (**C**). When successful puncture was confirmed, the dilator tip was forced into the sac and the SL0 sheath was exchanged for an 8F 30 cm Flexor sheath (Cook Medical, Bloomington, Ind) and a 5 Fr 65 mm catheter, which was used for the catheterization of the contributing vessel (**D**). Selective coil embolization of the contributing vessel was performed (**E**) and was followed by non-selective embolization of the nidus (**F**). After embolization, a final angiography was performed (**G**). Venous access sites were closed by manual compression and arterial access sites with either manual compression or a closure device. A pre-discharged CTA was performed to confirm EL 2 exclusion and to use as a baseline for further diameter follow-up (**H**).

The patients were managed under local or general anesthesia, according to objective criteria after anesthesiologist evaluation and the scheduled procedure (TCE in addition to transarterial IMA embolization or iliac interventions). The technique has been previously described in detail (Figure 1) [14]. No significant modifications were made during the last decade. 

Regarding the applied embolic materials, coils (Nester and Mreye, Cook Medical, Bloomington, IN, USA and VortX, Boston Scientific, Marlborough, MA, USA), in addition to liquid agents (1:1 mixture of n-butyl-2-cryanoacrylate and ionized oil), were used. Other embolic materials including vascular plugs (Amplatzer Vascular Plug, St. Jude Medical, St. Paul, MN, USA) were used depending on endoleak characteristics and the surgeon’s preferences. Selective embolization was attempted in all cases, and regardless of its success, it was followed by a non-selective embolization of the sac. The time spent to achieve a selective embolization was at the discretion of the operator and ranged usually between 15 and 30 min. Completion angiography and cavography were performed at the end of the procedure.

### 2.4. Follow-Up after TCE 

A pre-discharge CTA or DUS was performed to confirm or exclude the presence of EL 2. In the case that an EL 2 was present or pre-discharge imaging was not performed, the patient underwent evaluation at 3 to 6 months after the TCE. If an EL 2 was not detected at the pre-discharge imaging, the patient was evaluated at 6 months and yearly thereafter. Follow-up imaging was performed with CTA, native CT, DUS, CEUS, or a combination of imaging modalities depending on the findings and patient’s renal function. 

### 2.5. Definitions

Technical success was considered the successful transcaval puncture of the aneurysm sac and the deployment of the embolic material into the sac or contributing vessels. Cases scheduled to be managed with combined transarterial embolization of the IMA and TCE were not considered failures. If a TCE attempt was abandoned, and a transarterial embolization followed during the same session, the case was considered failed.

Clinical success was defined as the freedom for EL 2-related reinterventions, including open conversion, during follow-up. The indications for EL 2 reinterventions were failed previous TCE or recurrent EL 2 with sac expansion ≥5 mm. Reinterventions for other types of endoleaks (I or III) were not considered as clinical failures. 

Sac behavior was assessed using a threshold at 5 mm (stable (within 5 mm), shrinkage (decrease ≥ 5 mm), and growth (increase ≥ 5 mm)), comparing the pre-TCE diameter to the diameter of the latest available follow-up [15].

Selective embolization was the catheterization and coiling of at least one in- or outflow vessel contributing to EL 2 while non-selective embolization was the deployment of embolic material within the endoleak nidus.

Other outcomes were assessed using the Society for Vascular Surgery reporting standards, when applicable [16].

### 2.6. Outcomes

The primary outcomes were technical success and clinical success during follow-up. The secondary outcomes were considered the peri-operative mortality, major adverse events, and reinterventions. 

### 2.7. Statistical Analysis

Normally distributed continuous data were reported as mean ± standard deviation and non-normally distributed data as median values with range and interquartile range (IQR). Categorical data were expressed as absolute numbers and percentages. The Chi-square test was used for the comparison of categorical data. Independent two-sample *t* tests were used for normally distributed continuous variables, and the Mann–Whitney U test for non-normally distributed continuous and ordinal variables. Kaplan–Meier estimates were performed to assess follow-up outcomes including clinical success, survival, and freedom from any reintervention. The initial endovascular treatment (EVAR vs. f/bEVAR), type of embolization (selective vs. non-selective), as well as the patient’s treatment with anticoagulants were assessed as confounders for follow-up outcomes through Cox regression models. No correction for multiple hypothesis testing was applied. The sample size was allowed to vary based on the analysis and no imputation of missing data was performed, as it was infrequent for both categorical and continuous variables (<5%). The *p* value was considered significant when it was <0.05 (two-tailed hypothesis). Statistical analysis was performed by SPSS 29.0 for MacOS (Sonoma 14.5) software (IBM Corp, Armonk, NY, USA).

## 3. Results

### 3.1. Patient Cohort

In total, 43 patients underwent TCE for EL 2. The mean age was 75.1 ± 6.0 years and 90.7% (39/43) were males. The patients’ comorbidities are presented in Table 1. One patient had spinal cord ischemia (SCI) after a previous complex endovascular repair. Eight patients (18.6%) had atrial fibrillation; all were on anticoagulant treatment [two with vitamin K antagonists and six with direct oral anticoagulants (DOACs)]. The median American Society of Anesthesiologists score was 3 (IQR 0, range 2–4) and 81.4% (35/43) of patients had an ASA score ≥ 3.

Regarding the patients’ aortic history, twenty-nine (67.4%) were previously managed with EVAR, six (14.0%) were managed with fEVAR, and eight (18.6%) with bEVAR. Aneurysm extension is presented in Table 2. The mean aortic diameter before EVAR or f/bEVAR was 60.8 ± 9.4 mm. Six patients had a previous coil embolization for EL 2: five with a transarterial approach and one with para-endograft access. The median time from endovascular aortic repair to TCE was 35 months (IQR 31, range 7–120 months).

### 3.2. Pre-Operative Imaging Findings

All patients were evaluated with CTA pre-TCE; in five, the diagnosis of EL 2 was not clear and was finally confirmed using DUS. In total, 16 (40.0%, 16/43) patients underwent an additional ultrasound pre-TCE (11 with contrast). The mean aneurysm diameter before TCE was 67.4 ± 9.2 mm.

### 3.3. Intra-Operative Details

Nine patients (20.9%) were managed under local and the remaining under general anesthesia. All patients had a right common femoral vein (RCFV) access and a left (LCFA) or right common femoral artery (RCFA) access. For the RCFV access, an 8 Fr sheath was used (median 8, IQR 0, range 7–10) while for the femoral artery access, smaller diameter sheaths were preferably used (LCFA: median 4, IQR 1, range 4–8 Fr, and RCFA: median 5 Fr, IQR 2, range 4–14 Fr). In one case, where a 14 Fr sheath was introduced, a distal limb extension was also performed. In two cases, a steerable sheath was inserted to permit appropriate contributing vessel catheterization.

Intra-operative angiography confirmed the presence of contributing vessels related to EL 2 in all but five cases (11.6%, 5/43). In these five patients, the endoleak nidus was visible but no contributing vessel could be detected, and a non-selective embolization was decided. In five cases, the IMA was contributing to EL 2 formation, while in seven cases, a concomitant IMA and lumbar artery endoleak was detected. Lumbar arteries contributed to EL 2 in 28 cases (65.1%).

Non-selective coiling of the sac was performed in 22 cases (51.2%) while in the remaining cases, a combination of selective and non-selective coiling was applied (48.8%, 21/43). Coils were used in all cases. The median number of coils per patient was eight (IQR 5, range 2–36 coils). In 25 patients, histoacrylic glue was also used for sac embolization. The mean volume of liquid agent/patient was 3.0 ± 1.6 mL. In one patient, an additional vascular plug was used while in four cases, relining of the right limb was performed. One patient underwent a concomitant stenting of the external iliac artery.

Technical success was achieved in 42 patients (97.7%). In one patient, no sac access was achieved. Completion angiography confirmed the exclusion of the endoleak in 27 patients (62.8%). The mean operative time was 134.8 ± 38.3 min, the volume of contrast used was 82.7 ± 24.2 mL, the radiation exposure was 232 ± 105 Gy·cm^2^, and the fluoroscopy time was 34.2 ± 5.2 min.

### 3.4. 30-Day Outcomes

No death was recorded at 30 days. One patient presented with a retroperitoneal hematoma after TCE and was managed conservatively. This was the only detected MACE, leading to a 2.3% MACE rate. No patient presented with signs of SCI or acute kidney injury. The pre-discharge creatinine was 1.1 ± 0.3 mg/dL and the estimated glomerular filtration rate was 69.3 ± 13.8 mL/min/1.73 m^2^. None of the patients needed a reintervention within 30 days after TCE. The median hospital stay was 2 days (IQR 1, range 1–8 days) and all patients were discharged home.

Pre-discharge imaging was performed in 27 patients (62.7%). Eleven patients underwent a CTA, five patients underwent a combination of CTA and DUS, and the remaining eleven cases underwent only DUS. An EL 2 was detected in nine patients (20.9%). In all cases, the endoleak, if detectable, seemed to be smaller than the initial pre-TCE imaging.

### 3.5. Follow-Up Findings

The median follow-up was 36 months (IQR 41.5, range 1–108 months). Three patients (7.0%) were lost to follow-up. One patient died 48 months after the TCE procedure due to hemothorax after positioning a dialysis catheter. No other death was recorded. The estimated survival was 92% (standard error; SE: 7.4%) at 48 months of follow-up (Supplementary Figure S1).

The most used imaging modality during follow-up was CTA, in 31 cases (77.5%), while the remaining cases were followed with DUS. Seven patients had an endoleak detected during follow-up. Among them, three (7.5%, 3/40) had an EL 2; two were related to the lumbar arteries and one to the IMA. The remaining patients had an EL 1; one type 1a, one type 1b, and two type 1c. During follow-up, eleven patients presented with sac increase (27.5%). Among the remaining patients, 18 (45.0%) presented with a stable sac and 11 (27.5%) with sac decrease.

**Figure 2:**
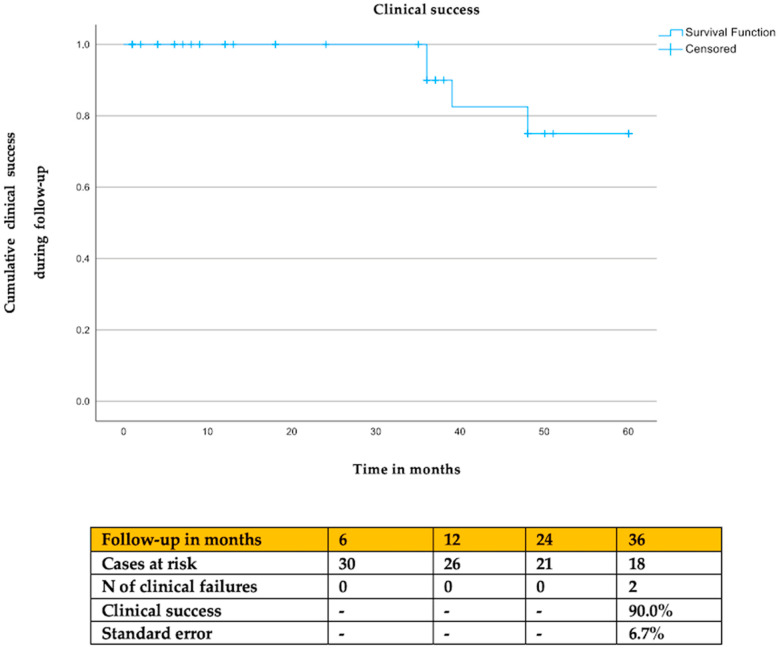
The estimated clinical success during follow-up. Clinical success was considered the absence of EL 2-related reinterventions.

**Figure 3:**
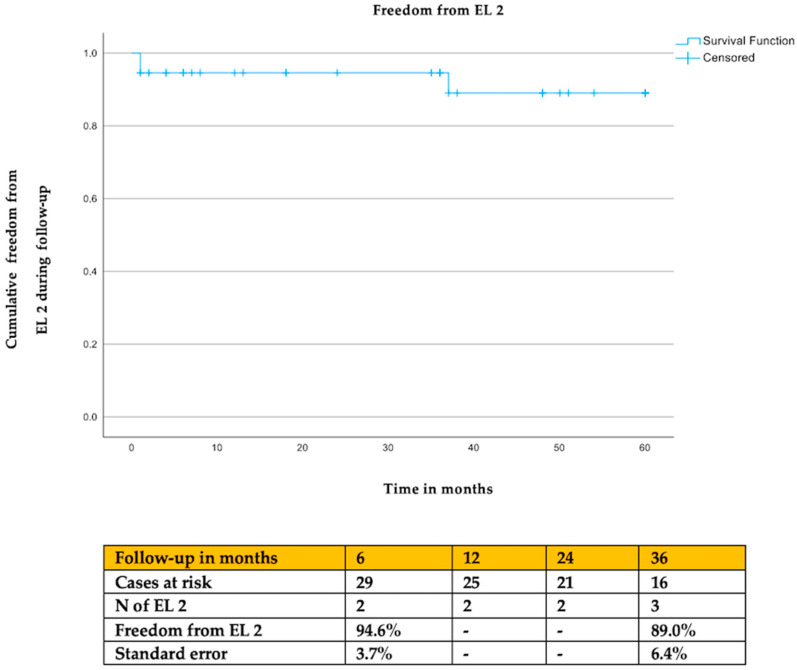
The estimated freedom from EL 2 during follow-up.

The estimated clinical success was 90.0% (SE: 6.7%) and the freedom from EL 2 was 89.0% (SE 6.4%) at 36 months of follow-up (Figure 2 and Figure 3). Cox regression analysis showed that the type of embolization (*p* = 0.91), type of previous endovascular aortic repair (*p* = 0.97), and use of anticoagulants (*p* = 0.83) had no impact on clinical success or freedom from EL 2 (type of embolization: *p* = 0.96; type of previous endovascular repair: *p* = 0.49; anticoagulants: *p* = 0.19).

In total, twelve patients underwent reintervention of any type, with seven needing proximal extensions for EL 1a or loss of sealing. The median estimated time of reintervention after TCE was 36 months (IQR 3–84 months). Three patients underwent four redo-embolizations, with two transarterial and two transcaval. Among them, one patient after two failed embolization attempts, underwent open conversion and lumbar artery ligation for a persistent EL 2. Two additional open conversions were performed; one after failed TCE and one due to aneurysm sac progression, despite successful embolization (Table 3).

## 4. Discussion

This retrospective analysis of 43 patients managed with TCE for EL 2 showed high technical success, related to low post-operative adverse event rates and no mortality, confirming the safety and effectiveness of TCE. The clinical success was estimated at 90.0% at 36 months of follow-up, showing that TCE, when applicable, may provide a viable alternative for EL 2 management. The freedom from EL 2 was high at 36 months of follow-up. However, 7% of patients needed an open conversion due to persistent endoleak or sac expansion. These findings confirm previous smaller cohorts’ findings, with high technical and acceptable clinical success [14,17]. The nature of previous aortic repair, EVAR vs. f/bEVAR, and the type of embolization, selective vs. non-selective, did not affect follow-up outcomes, showing the extend of disease and successful selective vessel catheterization may not affect the efficacy of TCE [14].

Embolization for EL 2 is a safe procedure, with null peri-operative mortality, low morbidity, and high technical success rates up to 98% [14,17,18]. Previous systematic review and meta-analysis data showed that translumbar embolization provides a safer and more effective option compared to the transarterial approach [19,20]. However, its outcomes are affected by factors, such as the use of antiplatelet treatment and aneurysm sac size [21]. To our knowledge, data comparing any other approach with TCE are not available. Previous non-comparative data on TCE confirmed a technical success rate up to 91.4% in 128 TCE attempts [12]. The type of previous endovascular repair as well as the application of selective embolization did not seem to affect the technical success rates, as described also in the current analysis [13,22].

The clinical success rate was estimated at 90.0% at 36 months of follow-up. To our knowledge, this is the longest available follow-up of TCE outcomes compared to previous studies [14]. Clinical success was independent from pre- and intra-operative parameters. However, previous studies showed that EL 2 occurrence, sac expansion, and need for reintervention are all affected by anatomic and hemodynamic factors [14,22]. Patients with multiple contributing vessels related to EL 2 and inflow–outflow patterns, as well as the presence and distribution of endoluminal thrombus before repair, may lead to persistent EL 2 and subsequently, multiple reinterventions, and even open conversions, to achieve sac exclusion [1,4,23,24]. In addition, hemodynamic alterations after a successful embolization may lead to the evolution of new endoleaks, including type 2.

Data on EL 2 after complex endovascular procedures are limited [3]. A recent study of 132 patients managed for complex aortic aneurysms, showed that EL 2 was detected in 27% of patients at pre-discharge imaging with only one in three of them resolving spontaneously [3]. Once again, parameters such as the presence of thrombus, number of patent lumbar arteries, and use of antiplatelet treatment were independently related to EL 2 evolution [3]. Previous data showed that complex anatomy and repairs may affect the need for reintervention after TCE, potentially due to the higher number of contributing vessels in supra-renal aortic pathologies [14]. However, no statistically significant difference was achieved in that analysis while the available data at that moment underpowered any comparison [14]. In the current cohort, f/bEVAR was not detected as a worsening factor for clinical success. However, the limited number of adverse events and the small sample size should be once again acknowledged.

The use of antiplatelet treatment in patients managed with EVAR or f/bEVAR is currently indicated according to the latest guidelines, as a measure not only to decrease cardiovascular morbidity and mortality but also to preserve target vessel patency [25,26,27]. Previous studies showed that antithrombotic factors were related to EL 2 persistence, especially in patients managed with vitamin K antagonists [3,28]. Recent comparative data on patients under combined treatment with acetylsalicylic acid and anticoagulants vs. patients under monotherapy with aspirin showed that anticoagulants were independently related to lower rates of sac regression and a higher risk for EL 2 formation [29]. However, the reintervention rate between groups was similar [29]. No difference between vitamin K antagonists and direct oral anticoagulants regarding EL 2 evolution has been detected [29]. In the current study, the use of anticoagulation did not affect the clinical success rate of TCE, probably showing that the development and persistence of EL 2 may be multifactorial rather than directly related to the antithrombotic treatment. Any comparison between vitamin K antagonists (two patients) and DOACs (six patients) was not possible due to the limited number of cases but it is a topic of increasing interest. A multidisciplinary approach, balancing the risks of cardiovascular morbidity and endoleak formation with sac expansion, is mandatory to provide the optimal individualized management in patients needing anticoagulant treatment and endovascular aortic repair.

It should be noted that seven patients (17.5%) needed a reintervention for EL 1a or loss of sealing during follow-up. This finding confirms that despite EL 2 being considered a benign condition, sac enlargement may lead to more severe complications and even more complex future procedures [6,30,31]. Recent data showed that despite successful EL 2 embolization, almost 20% of patients presented with signs of proximal and distal sealing loss and required further reintervention [6]. These findings highlight the need for appropriate and meticulous imaging surveillance with sac diameter evaluation in order to prevent future instability, complications, and reinterventions. Pre-emptive embolization to prevent EL 2 has been applied with encouraging outcomes, including significantly lower rates of sac expansion and EL 2-related reinterventions [32]. However, data on cost-effectiveness, radiation exposure, and incidence of aortic rupture are limited or completely lacking [10,32].

### Limitations

The retrospective nature and limited sample size are the main parameters affecting the findings of this analysis. The sample size (forty-three patients), though it may be considered large, represents a decade of experience in TCE for EL 2 in a referral high-volume aortic center and leads to a rate of four TCEs per year. Three patients were lost to follow-up, leading to a 7% rate, and potentially affected our outcomes. Various aortic pathologies were managed with various techniques, types, and designs of endografts, increasing the heterogeneity of the cohort. However, complex aortic procedures did not affect the mid-term outcomes. A variety of materials were used, hampering for the moment any comparison, while the decision making for material selection was at the discretion of the operator and the availability at the moment of TCE. Different imaging modalities were used for both EL 2 diagnosis and follow-up after embolization. Despite the recognized significance of CEUS in these cases, the modality was not always available to our service, mainly related to the person performing the ultrasound. In addition, the presence of coils in the aneurysm sac potentially affected the diagnosis of persistent EL 2. Future studies, especially of comparative nature, are needed to determine the durability and effectiveness of the technique.

## 5. Conclusions

TCE was related to high technical success and limited peri-operative adverse events in well-selected cases, regardless of the type of initial endovascular aortic repair. Clinical success was encouraging with reinterventions for EL 2 affecting 10% of patients within the initial 36 months of follow-up. Open conversions were performed in 7% of cases.

## Figures and Tables

**Table 1:** Previous medical history of patients managed with TCE for EL 2.

Medical History	N of Patients (%)
Coronary artery disease	19 (44.2)
Atrial fibrillation	8 (18.6)
Chronic heart failure	3 (7.0)
Coronary–aortic bypass graft	5 (11.7)
Percutaneous coronary intervention	11 (25.6)
Hypertension	35 (81.4)
Dyslipidemia	18 (41.9)
Smoking	13 (30.2)
COPD	8 (18.6)
Diabetes mellitus	9 (20.9)
Chronic renal disease	17 (39.5)
Creatinine at admission (mg/dL)	1.19 ± 0.3
eGFR with MDRD (mL/min/1.73 m^2^)	65.3 ± 15.8
Stroke	3 (7.0)
Spinal cord ischemia	1 (2.3)
Genetic aortic syndrome	1 (2.3)
Peripheral arterial disease	4 (9.3)

COPD: chronic obstructive pulmonary disease; eGFR: estimated glomerular filtration rate; MDRD: modification of diet in renal disease; N: number.

**Table 2:** Previous aortic history of patients managed with TCE for EL 2.

Aortic Aneurysms Extend	N of Patients	Type of Repair
Infra-renal	29 (67.4)	29 EVAR
Juxta-renal	6 (14.0)	6 fEVAR
Para-renal	1 (2.3)	1 bEVAR
Thoracoabdominal	7 (12.3)	7 bEVAR
Type I	0 (0.0)	-
Type II	3 (7.0)	-
Type III	0 (0.0)	-
Type IV	4 (9.3)	-

EVAR: endovascular aortic aneurysm repair; bEVAR: branched endovascular aortic aneurysm repair; fEVAR: fenestrated endovascular aortic aneurysm repair.

**Table 3:** Forty patients completed follow-up. Among them, twelve patients underwent a secondary reintervention, with seven for proximal sealing loss.

Reinterventions	N of Patients (%)
Proximal extension with f/bEVAR	7 (17.5)
Due to EL 1a	5 (12.5)
Distal extension	3 (7.0) *
Iliac branch device	1 (2.5)
Redo-embolization	4 (10.0) **
Transarterial	2 (5.0)
Transcaval	2 (5.0)
Open conversion	3 (7.0)

EL: endoleak; f/bEVAR: fenestrated/branched endovascular aortic repair. * All three patients also received a proximal extension; ** One patient underwent two redo-embolizations.

## Data Availability

The data presented in this study are available on reasonable request from the corresponding author.

## Supplementary Materials

The following supporting information can be downloaded at: https://www.mdpi.com/article/10.3390/jcm13123578/s1, Figure S1: The estimated survival of patients managed with TCE for EL 2.
