# Supplementary material for: Early and Mid-Term Outcomes of Transcaval Embolization for Type 2 Endoleak after Endovascular Aortic Repair
Source: J Clin Med. 2024 Jun 18;13(12):3578. doi: 10.3390/jcm13123578 (PMC11204610; doi:10.3390/jcm13123578)
Supplement: Supplementary file 1 [file jcm-13-03578-s001.zip › jcm-3047395-supplementary.pdf]

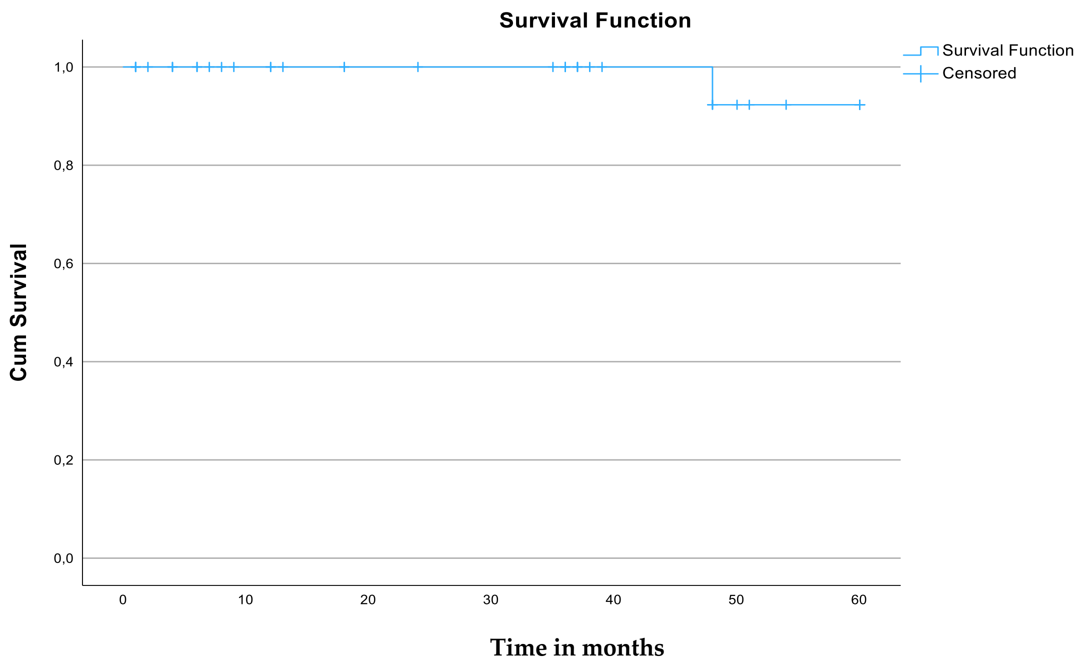

Follow-up in months	12	24	36	48
Cases at risk	26	21	19	12
N of deaths	0	0	0	1
Survival	-	-	-	92.3%
Standard error	-	-	-	7.4%

Supplementary Figure S1. The estimated survival of patients managed with TCE for EL 2.
